# Supplementary material for: Lupus Anticoagulant Positivity as a Risk Marker for Hemolytic Anemia in Patients with APS
Source: Medicina (Kaunas). 2025 Jul 28;61(8):1364. doi: 10.3390/medicina61081364 (PMC12388202; doi:10.3390/medicina61081364)
Supplement: Supplementary file 1 [file medicina-61-01364-s001.zip › medicina-3733817-supplementary.pdf]

Supplement S1. Comparison of Clinical and Laboratory Parameters Between AIHA+ and AIHA− Patients

Variable	AIHA+ (n=57)	AIHA− (n=289)	P-value
Thrombosis (%)	63.2%	40.8%	0.0031
Hemoglobin Nadir (g/dL)	8.66 ± 0.84	10.15 ± 1.18	<0.0001
PLT Nadir (×10^9/L)	111.43 ± 33.65	133.13 ± 33.30	<0.0001
ANA Positivity (%)	82.5%	52.9%	0.0001
C3 Nadir (mg/dL)	69.59 ± 15.22	86.25 ± 19.89	<0.0001
C4 Nadir (mg/dL)	12.43 ± 3.99	15.18 ± 5.01	<0.0001
LAC Positivity (%)	84.2%	59.8%	0.0002
Triple aPL Positivity (%)	61.4%	30.4%	0.0003
Immunomodulating Therapy (%)	75.4%	50.8%	0.0005

Supplement S2. Comparison of AIHA Prevalence Between SLE and Non-SLE Patients

Group	AIHA Present (n, %)	AIHA Absent (n, %)	Total (n)	p-value
SLE	20 (23.3%)	66 (76.7%)	86	0.074
Non-SLE	37 (14.2%)	223 (85.8%)	260	
